# Five-Axis Micro Ball-End Milling Force Prediction for Micro Curved-Surface Parts

**DOI:** 10.3390/mi17080961

**Published:** 2026-08-15

**Authors:** Zhenghu Yan, Yicheng Yang, Shuai Wang, Chenxi Yang, Ruisi Qin

**Affiliations:** 1 School of Mechatronic Engineering, Xi’an Technological University, Xi’an 710021, China; 13572207092@163.com (Y.Y.); 15002980572@163.com (C.Y.); qrscf123@163.com (R.Q.); 2 Xi’an Jingdiao Software Technology Co., Ltd., Xi’an 710119, China; wangshuai@jingdiao.com

**Keywords:** cutting force, five-axis, ball end micro milling, cutter–workpiece engagement, instantaneous undeformed chip thickness

## Abstract

Micro curved-surface parts are widely used in the aerospace, defense, biomedical, and automotive industries, and their growing adoption imposes increasingly stringent performance requirements. Five-axis micro-milling can achieve precision machining of parts with complex shapes. In the micro-milling process, the cutting force is a critical parameter, as it is the main factor causing machining deformation, vibration, and tool wear. Therefore, this study develops a prediction model for five-axis micro-milling forces in the machining of micro complex curved-surface parts. First, four coordinate systems were established for the five-axis milling process, and the transformation relationships among them were derived. A cutter–workpiece engagement (CWE) extraction method based on solid modeling was also introduced. Then, an instantaneous undeformed chip thickness (IUCT) model was established, taking into account tool runout, elastic recovery of the machined surface, minimum chip thickness, and the local radius of the micro ball-end mill. On this basis, a five-axis micro-milling force prediction model was developed. Finally, five-axis micro-milling experiments were conducted on a micro-impeller and a micro-spherical part, and the cutting forces at different cutter location (CL) points were measured. For the micro-impeller blade, the average percentage errors in the X, Y, and Z directions at all selected CL points were below 11.2%; for the micro-spherical part, the corresponding errors were below 14.4%. These results show good agreement between the predicted and measured values, verifying the effectiveness of the proposed model.

## 1. Introduction

With the rapid development of micro-manufacturing technology, micro curved-surface parts have found increasingly widespread application in the aerospace, defense, biomedical, and automotive industries. Five-axis micro-milling is an effective technique for producing such parts, as it enables the precision machining of complex three-dimensional geometries from a wide range of materials with high flexibility. In the five-axis micro-milling process, the milling force is a critical process parameter that provides the analytical basis for other machining-related physical quantities, such as machining vibration and force-induced deformation. Therefore, establishing a milling force prediction model for the five-axis micro-milling of micro curved-surface parts is of great significance for improving both machining efficiency and precision.

To date, notable advances have been achieved in cutting force modeling for five-axis milling of complex surfaces, establishing a sound foundation for the physical simulation of CNC machining. The extraction of cutter–workpiece engagement (CWE) and the calculation of instantaneous uncut chip thickness (IUCT) constitute fundamental prerequisites for developing a five-axis milling force model. Consequently, extensive research efforts have been devoted to CWE extraction and IUCT computation. To improve the prediction accuracy of five-axis milling forces, Qin et al. [1] proposed a CWE boundary identification algorithm under variable cutter orientations, and an enhanced Z-map method and a simplified IUCT model were developed to address the difficulty in predicting cutting forces caused by the simultaneous variations in CWE and IUCT in ball-end milling. Experimental validation demonstrated satisfactory cutting force prediction accuracy. Zhou et al. [2] determined the actual CWE region through an upper–lower boundary optimization criterion, and formulated an IUCT model based on outward normal vectors of differential points that incorporates static deformation, cutter runout, and tool orientation variation effects. The proposed methodology resolves the modeling challenges of milling forces associated with the concurrent variation in CWE and IUCT in the multi-axis machining of thin-walled complex surfaces. Regaieg et al. [3] proposed an enhanced analytical model to characterize the effects of lead and tilt angles on CWE and IUCT in five-axis ball-end milling, thereby improving the accuracy of cutting force prediction under variable tool orientations. Zhuang et al. [4] proposed a cutting force prediction model for multi-axis ball-end milling based on a novel uncut chip thickness (UCT) formulation. The proposed UCT model explicitly accounts for the effects of lead and tilt angles. The validity of the developed model was verified through finite element method (FEM) simulations and multi-axis milling experiments. Duan et al. [5] developed a prediction model that accounts for force–deflection coupling in five-axis milling with fillet-end cutters. An iterative method was employed to resolve the feedback relationship between cutting force and cutter deflection, thereby enhancing the prediction accuracy. Aydın et al. [6] proposed a cutting force prediction methodology for side milling based on the force distribution along the cutter axis. By experimentally identifying the force distribution and calibrating the milling coefficients to account for the effects of cutter geometry and cutting parameters, the method enhances prediction accuracy relative to average-force approaches that disregard the axial force distribution of helical flutes. Guo et al. [7] developed a cutting force prediction model for five-axis ball-end milling of free-form surfaces that accounts for the mesoscale size effect. A novel modified expression for dislocation density was introduced, and dedicated calibration experiments were designed to identify the dislocation density correction coefficient. Consequently, the prediction accuracy of milling forces under micrometer-scale uncut chip thicknesses in macro-scale milling was improved. To improve the calculation efficiency of five-axis milling forces, Tang et al. [8] proposed a geometric simulation method for five-axis milling based on a GPU-accelerated tri-dexel model, enabling high-fidelity determination of the CWE and IUCT for ball-end cutters with helical flutes. Ge et al. [9] proposed an analytical IUCT calculation method that accounts for cutter runout. An explicit parametric IUCT expression was derived from this method for cutting force prediction, thereby significantly reducing the computational cost. Tian et al. [10] established a cutting force prediction model for variable-pitch ball-end cutters in five-axis milling. By employing planar projection of the CWE and coordinate system transformation, the CWE under a fixed tool axis is first determined and subsequently mapped to arbitrary tool orientations, enabling the prediction of the engagement zone and cutting forces under dynamic tool orientations. The aforementioned investigations have laid a solid foundation for the prediction of cutting forces in five-axis milling of complex curved workpieces. Nevertheless, they remain largely confined to macro-scale components, and the milling forces of micro complex-surface parts under five-axis machining conditions have scarcely been addressed.

Regarding the prediction of cutting forces in micro-component machining, Bao et al. [11] established a micro-milling force model based on the trochoidal motion of tool tips, incorporating the effect of cutter runout. Altintas et al. [12] predicted cutting forces by employing a slip-line field model coupled with a material constitutive model and a friction coefficient. Jun et al. [13] formulated a micro-milling force model that incorporates multiple cutting phenomena, including the ploughing effect, elastic recovery of machined surfaces, effective rake angle, and flank friction. Their investigation revealed that the cutting-edge radius exerts a pronounced influence on the ploughing effect, frictional force components, and effective rake angle. Sun et al. [14] discretized the micro ball-end mill into cutting-edge micro-elements along the axial direction. The elemental unit cutting force was determined through finite element simulation and regression analysis, and the total micro-milling force was predicted by integrating these elemental forces. Gao et al. [15] proposed an IUCT model that simultaneously accounts for tool runout, tool flank wear, and tool edge radius. Based on this model, they further developed a cutting force model for the micro-end-milling process, and its validity was experimentally demonstrated through micro-milling tests. Zhang et al. [16] investigated the micro-cutting mechanism and cutting force evolution of 7075 aluminum alloy, with particular emphasis on the influence of cutting-edge radius, depth of cut, and feed rate. The aforementioned studies have laid a solid foundation for the advancement of micro-milling force models and have provided reliable references for subsequent research. However, the majority of these studies remain confined to three-axis machining with flat-end or ball-end micro-mills, primarily addressing parts with simple geometries and slot milling experiments.

In summary, existing studies on milling forces have predominantly focused on the five-axis milling of conventional-scale complex parts and the three-axis micro-milling of simple-geometry parts. Conventional-scale five-axis milling force models neglect the size effects associated with the reduced workpiece and tool dimensions, and are therefore inapplicable to five-axis micro-milling. Meanwhile, existing micro-milling force models have primarily been developed for the three-axis milling of simple planar parts, rarely addressing micro curved-surface parts or accounting for the CWE variation induced by the changing tool axis orientation. Accordingly, the original contribution of this study lies in bridging these two gaps: we propose a five-axis micro-milling force prediction model that simultaneously accounts for tool runout, elastic recovery of the machined surface, the minimum chip thickness, and the local curvature radius of the micro ball-end mill. The validity of the proposed model is experimentally verified through five-axis micro-milling tests conducted on a micro-impeller and a micro-spherical part.

The remainder of this paper is organized as follows. First, the coordinate system transformation for five-axis milling and the CWE extraction are introduced, followed by a detailed derivation of the IUCT. Second, a milling force prediction model for the five-axis micro-milling process is developed. Finally, the effectiveness of the proposed model was validated through five-axis micro-milling experiments on a micro-impeller and a micro-spherical part.

## 2. Method

### 2.1. Coordinate Transformations for Five-Axis Milling

**Figure 1:**
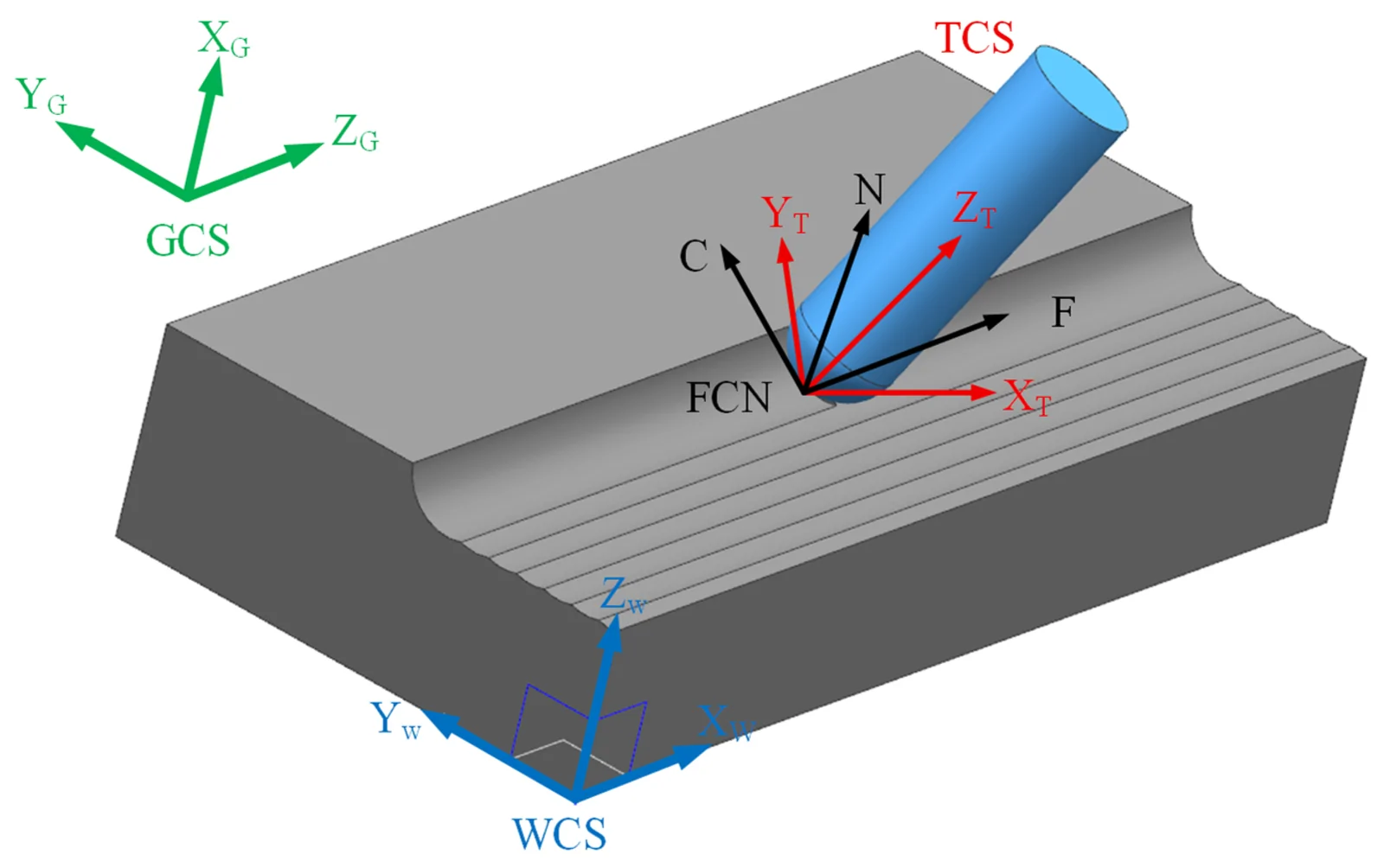
Coordinate systems in five-axis micro-milling.

To precisely characterize the instantaneous position and posture of the ball-end cutter during five-axis milling, four coordinate systems are adopted to define the tool state: the Global Coordinate System (GCS), the Workpiece Coordinate System (WCS), the Feed–Cross-Feed–Normal coordinate system (FCN, also known as the machining coordinate system), and the Tool Coordinate System (TCS), as illustrated in Figure 1.

In Figure 1, the GCS is defined as a fixed, right-handed Cartesian frame comprising the orthogonal axes X_G_, Y_G_, and Z_G_. The WCS is rigidly affixed to the workpiece. Throughout this study, the GCS is assumed to coincide with the WCS. The FCN is established by the feed vector $\vec{f}$, the cross-feed vector $\vec{c}$, and the normal-feed vector $\vec{n}$, with its axes denoted by F, C, and N, respectively. The origin of the FCN is positioned at the cutter tip of the ball-end mill. The TCS is obtained by rotating the FCN frame about the corresponding coordinate axis, with its origin coincident with that of the FCN. Since the GCS coincides with the WCS, three coordinate systems, namely the GCS, the FCN, and the TCS, are jointly employed to describe the complex tool-motion trajectories in the five-axis milling process.

During the five-axis milling process, the coordinate transformation between the FCN and the TCS is established based on geometric relationships. First, the FCN is rotated counterclockwise about the F-axis by an angle *β* to obtain an intermediate coordinate system, denoted as the X′Y′Z′ coordinate system. Subsequently, the intermediate coordinate system X′Y′Z′ is rotated about the Y′-axis by an angle *α* to yield the TCS. Here, *α* and *β* represent the lead angle and the tilt angle of the cutter, respectively.

The transformation from the GCS to the TCS is accomplished in two steps. First, the GCS is transformed into the FCN via a combined translation and rotation. The FCN is then rotated to yield the TCS [17]. The detailed coordinate transformation is described as follows:

(1) $$\left(X_{T},Y_{T},Z_{T}\right)=\left(F,C,N,1\right)T_{1}R_{1}R_{2}$$

(2) $$T_{1}=\left[\begin{array}{cccc} 1 & 0 & 0 & 0\\ 0 & 1 & 0 & 0\\ 0 & 0 & 1 & 0\\ -X_{T} & -Y_{T} & -Z_{T} & 1 \end{array}\right]$$

(3) $$R_{1}=\left[\begin{array}{cccc} F_{i} & C_{i} & N_{i} & 0\\ F_{j} & C_{j} & N_{j} & 0\\ F_{k} & C_{k} & N_{k} & 0\\ 0 & 0 & 0 & 1 \end{array}\right]$$

(4) $$R_{2}=\left[\begin{array}{cccc} \cos\alpha & 0 & \sin\alpha & 0\\ \sin\alpha \cos\beta & \cos\beta & -\cos\beta \sin\beta & 0\\ -\sin\alpha \cos\beta & \sin\beta & \cos\alpha \cos\beta & 0\\ 0 & 0 & 0 & 1 \end{array}\right]$$

Specifically, *T*_1_ and *R*_1_ denote the translation matrix and the rotation matrix from the GCS to the FCN, respectively, while *R*_2_ denotes the rotation matrix from the FCN to the TCS. The coordinates of the tool tip of the ball-end mill are defined as (*X_T_*, *Y_T_*, *Z_T_*). In addition, *F =* (*F_i_*, *F_j_*, *F_k_*), *C =* (*C_i_*, *C_j_*, *C_k_*)*,* and *N =* (*N_i_*, *N_j_*, *N_k_*) are the unit vectors corresponding to the three coordinate axes of the FCN frame.

### 2.2. Calculation of Tool Inclination Angles Based on Cutter-Location (CL) Files

**Figure 2:**
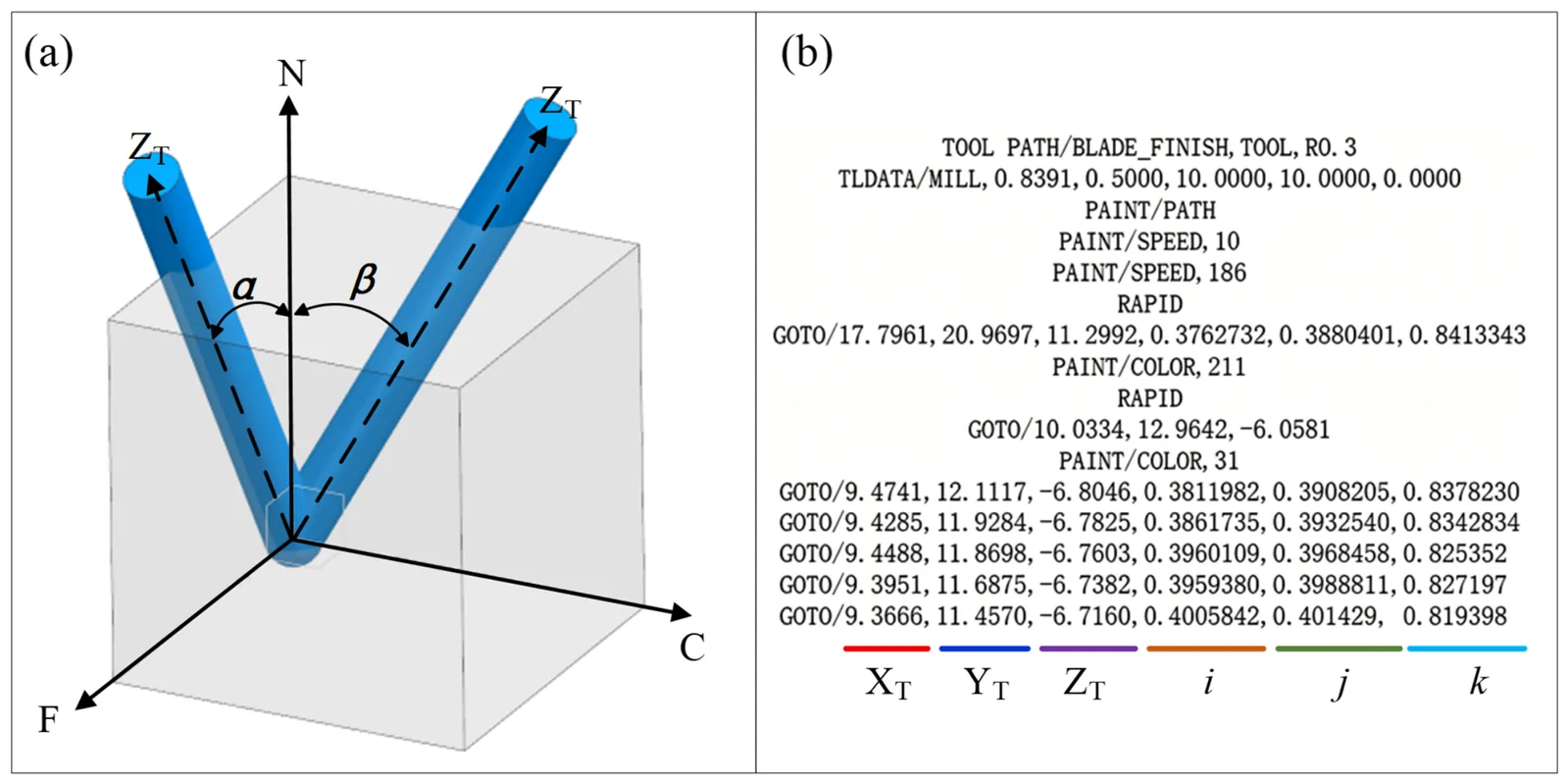
Schematic diagrams of tool tilt angle and lead angle and composition of CL file data. (**a**) Tool tilt angle and lead angle. (**b**) CL file data.

In the five-axis milling system, two critical angles are involved in addition to the four coordinate systems introduced above: the lead angle and the tilt angle, as shown in Figure 2a. The lead angle α is defined as the rotation angle of the tool axis about the cross-feed axis C, while the tilt angle β is defined as the rotation angle of the tool axis about the feed axis F. These two angles are used to determine the spatial orientation of the tool axis.

To accurately obtain the lead and tilt angles of the cutter during machining, the cutter location (CL) file is extracted from the UG NX 12.0 CAM software. The CL file primarily comprises the cutter tip coordinates and the tool axis vector. In most CAM software, the six numerical values following the GOTO command correspond to the three cutter tip coordinates and the three components of the tool axis vector, as shown in Figure 2b. Based on the cutter tip coordinates (*X_T_*, *Y_T_*, *Z_T_*) and the tool axis vector (*i*, *j*, *k*) extracted from the CL file, the lead angle *α* and the tilt angle *β* can be derived using inverse trigonometric functions as follows:

(5) $$\alpha =\arcsin\left(\frac{j}{\sqrt{i^{2}+j^{2}}}\right)$$

(6) $$\beta =\arcsin\left(\frac{i}{\sqrt{i^{2}+j^{2}}}\right)$$

### 2.3. Extraction of CWE Based on Solid Modeling Method

Three-dimensional solid models enable accurate geometric representation of the target structure. Therefore, the solid modeling method [18,19] is employed in this study to determine the CWE in five-axis micro-milling. Specifically, the geometric models of the tool and the workpiece are first constructed in a 3D CAD environment. Boolean operations are then performed between the tool and workpiece solids to simulate material removal, where the removed volume is represented by the intersection of the tool solid and the instantaneous workpiece solid. Finally, the CWE during the machining process is extracted.

During the five-axis micro-milling process, the cutter location (CL) changes continuously along the tool path, and the TCS changes accordingly. The FCN is determined from the CL file, and the TCS at the current CL point is computed via coordinate transformation. Once the TCS is established, the CWE at different CL points is obtained using the solid modeling method.

In the calculation of the CWE, three typical cutting conditions are generally involved: the first cut, slotting, and the following cut. The CWE extraction for each condition can be achieved through the solid modeling method.

### 2.4. Calculation of the Instantaneous Undeformed Chip Thickness

Accurate calculation of the instantaneous undeformed chip thickness (IUCT) is a prerequisite for precise prediction of five-axis micro-milling forces, while tool runout and elastic recovery of the machined surface are key factors influencing the IUCT. Therefore, both aspects are incorporated into the IUCT model established in the following sections.

During micro-milling, tool runout can lead to an uneven distribution of the IUCT, which further results in an uneven distribution of the milling force among the teeth. Moreover, compared with conventional milling, the ratio of tool runout to tool diameter in micro-milling is significantly higher. Therefore, the influence of tool runout on the IUCT cannot be neglected.

The trajectories of the *k*-th cutting tooth at time *t* and the (*k*-1)-th cutting tooth at time *t*′ can be expressed as follows:

(7) $$\left\{\begin{array}{c} x_{\left(k\right)}=R\sin(\omega t-\frac{2\pi k}{N_{t}})+R_{0}\sin(\omega t+\gamma_{0})+\frac{ft}{60}\\ y_{\left(k\right)}=R\cos(\omega t-\frac{2\pi k}{N_{t}})+R_{0}\cos(\omega t+\gamma_{0}) \end{array}\right.$$

(8) $$\left\{\begin{array}{c} x_{\left(k-1\right)}=R\sin(\omega t^{\prime }-\frac{2\pi (k-1)}{N_{t}})+R_{0}\sin(\omega t^{\prime }+\gamma_{0})+\frac{ft^{\prime }}{60}\\ y_{\left(k-1\right)}=R\cos(\omega t^{\prime }-\frac{2\pi (k-1)}{N_{t}})+R_{0}\cos(\omega t^{\prime }+\gamma_{0}) \end{array}\right.$$

where *R* is the radius of the tool in mm, *ω* is the spindle angular velocity in rad/s, *k* is the flute number, *N_t_* is the number of flutes, *R*_0_ is runout length in mm, *γ*_0_ is the run-out angle in rad, and *f* is the feed rate in mm/min.

**Figure 3:**
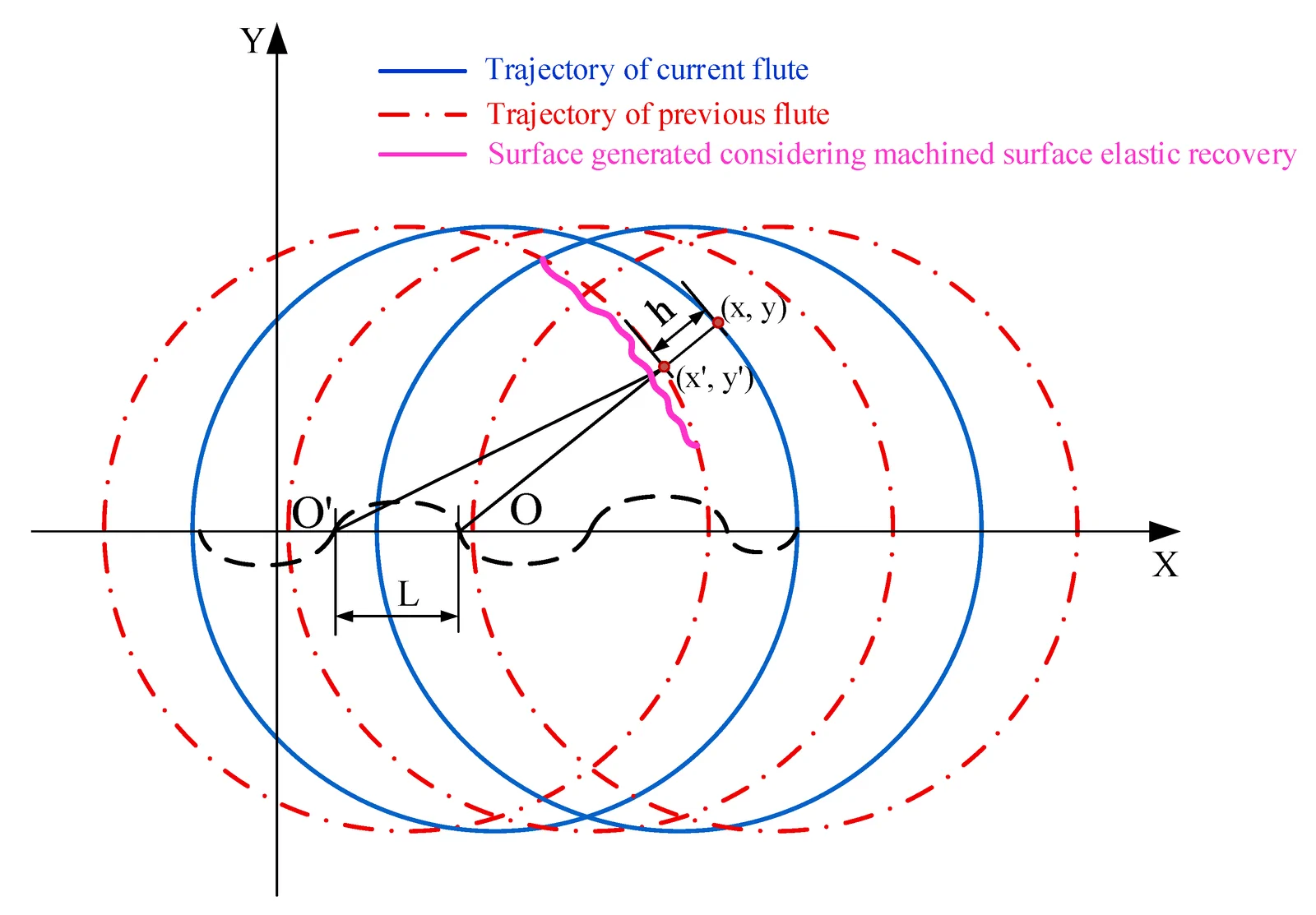
Tool trajectories of adjacent teeth with tool runout and machined surface elastic recovery. The black dashed line indicates the trajectory of the tool tip.

According to the geometric relationship shown in Figure 3, the IUCT in micro-milling considering tool runout can be calculated using the following equation [20]:

(9) $$h=R+L\sin\left(\omega t-\frac{2\pi k}{N_{t}}+\alpha_{0}\right)-\sqrt{R^{2}-L^{2}\cos^{2}\left(\omega t-\frac{2\pi k}{N_{t}}+\alpha_{0}\right)}$$

where *t* is the time (s). The distance *L* and the angle α_0_ can be given as follows:

(10) $$L=\sqrt{{\left(x_{O}-x_{O^{\prime }}\right)}^{2}+{\left(y_{O}-y_{O^{\prime }}\right)}^{2}}$$

(11) $$\alpha_{0}=\arctan\left(\frac{y_{O}-y_{O^{\prime }}}{x_{O}-x_{O^{\prime }}}\right)$$

The points *O* and *O*′ are the center positions of the tool at time *t* and *t*′, respectively.

(12) $$\left\{\begin{array}{c} x_{\left(O\right)}=R_{0}\sin(\omega t+\gamma_{0})+\frac{ft}{60}\\ y_{\left(O\right)}=R_{0}\cos(\omega t+\gamma_{0}) \end{array}\right.$$

(13) $$\left\{\begin{array}{c} x_{\left(O^{\prime }\right)}=R_{0}\sin(\omega t^{\prime }+\gamma_{0})+\frac{ft^{\prime }}{60}\\ y_{\left(O^{\prime }\right)}=R_{0}\cos(\omega t^{\prime }+\gamma_{0}) \end{array}\right.$$

Incorporating the elastic recovery height of the machined surface into the IUCT model is beneficial for improving the prediction accuracy of micro-milling forces. As illustrated in Figure 3, when the elastic recovery of the machined surface is considered, the trajectory of the previous tooth can be expressed as follows [21]:

(14) $$\left\{\begin{array}{c} x_{\left(k-1\right)}=(R-P_{e}\times h_{pre})\sin(\omega t^{\prime }-\frac{2\pi (k-1)}{N_{t}})+R_{0}\sin(\omega t^{\prime }+\gamma_{0})+\frac{ft^{\prime }}{60}\\ y_{\left(k-1\right)}=(R-P_{e}\times h_{pre})\cos(\omega t^{\prime }-\frac{2\pi (k-1)}{N_{t}})+R_{0}\cos(\omega t^{\prime }+\gamma_{0}) \end{array}\right.$$

where *P_e_* and *h_pre_* is the elastic recovery rate and height.

When considering both tool runout and elastic recovery of the machined surface, the IUCT in micro-milling can be rewritten as follows [21]:

(15) $$h\left(t\right)=\left(R-P_{e}\times h_{pre}\right)+Lsin\left(\omega t-\frac{2\pi k}{N_{t}}+\alpha_{0}\right)-\sqrt{{\left(R-P_{e}\times h_{pre}\right)}^{2}-L^{2}{cos}^{2}\left(\omega t-\frac{2\pi k}{N_{t}}+\alpha_{0}\right)}$$

In five-axis ball-end milling, the local radius *R*(*z*) of the cutter varies with the axial height. Accordingly, the IUCTs at different axial heights of the tool, denoted as *h*(*t*, *z*), also vary, and can be formulated as follows:

(16) $$h\left(t,z\right)=\left(R\left(z\right)-P_{e}\times h_{pre}\right)+L\sin\left(\omega t-\frac{2\pi k}{N_{t}}+\alpha_{0}\right)-\sqrt{{\left(R\left(z\right)-P_{e}\times h_{pre}\right)}^{2}-L^{2}\cos^{2}\left(\omega t-\frac{2\pi k}{N_{t}}+\alpha_{0}\right)}$$

where the local radius *R*(*z*) of the spherical segment of the ball-end mill varies with the axial height and can be calculated as follows:

(17) $$R(z)=\sqrt{R^{2}-(R-z{)}^{2}}$$

Unlike conventional five-axis milling, five-axis micro-milling should account for the size effect, which arises when the undeformed chip thickness becomes comparable to the cutting-edge radius. In this work, the size effect is manifested through the minimum chip thickness. When the IUCT is less than the minimum chip thickness (*h_min_*), the machining process is dominated by plowing and no chips are generated. In this case, the workpiece surface undergoes elastic recovery, forming a new machined surface. When the IUCT is greater than the *h_min_*, the machining process is dominated by shearing, resulting in chip generation and continuous removal of the workpiece material [21]. Then, the actual IUCT ha can be expressed as follows:

(18) $$h_{a}=\left\{\begin{array}{c} (1-P_{e})\times h;\;h\leq h_{min}\\ h;\;h>h_{min} \end{array}\right.$$

### 2.5. Five-Axis Micro-Milling Force Modeling

**Figure 4:**
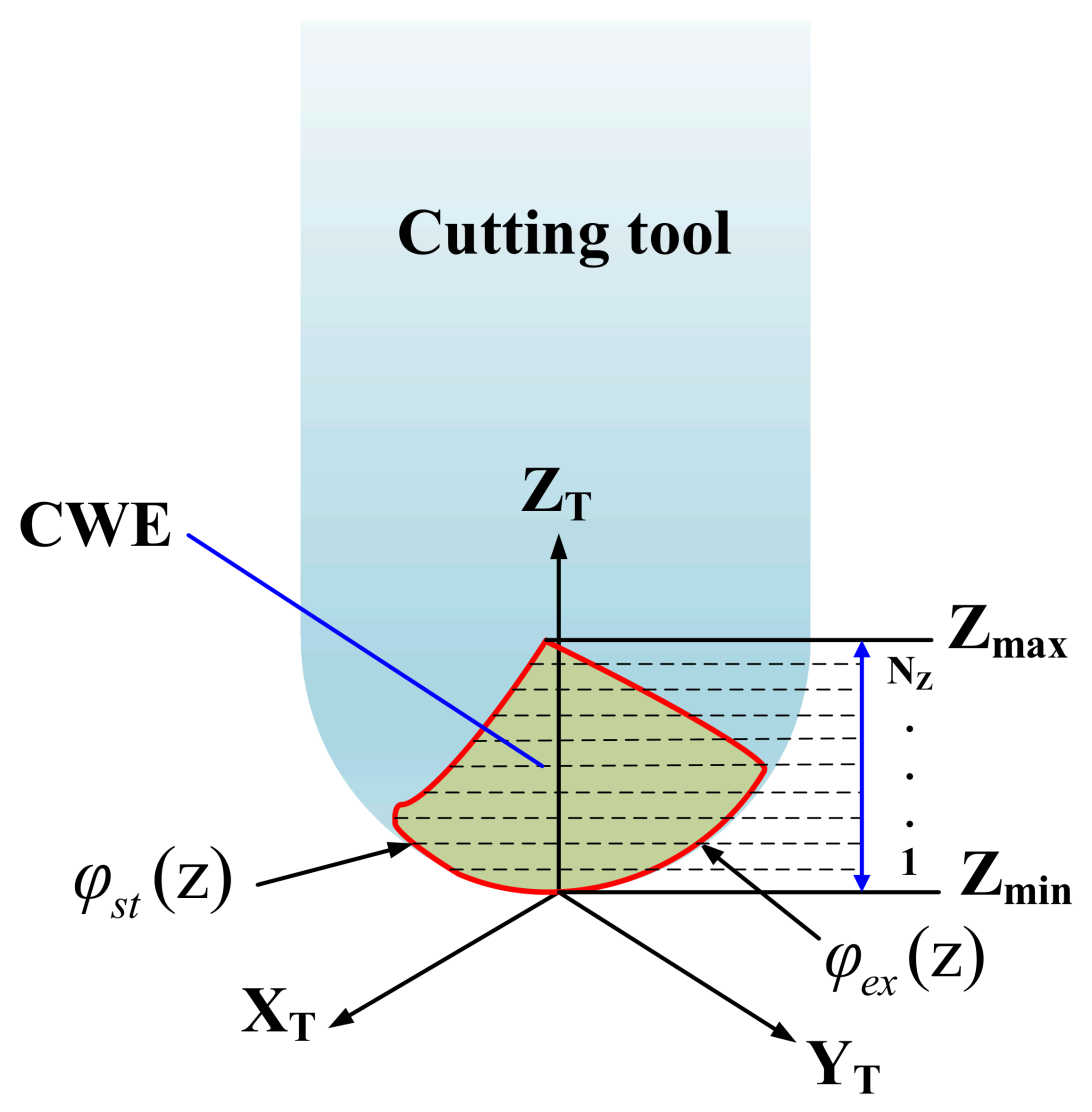
Axial discretization of CWE.

In the five-axis micro-milling process, the CWE varies with the tool position and orientation. At different axial heights of the cutting tool, the entry and exit angles of the cutting edges involved in cutting are different. Therefore, to predict the cutting force during five-axis micro-milling, the cutting edges are discretized into a series of elements along the axial direction based on the CWE, as shown in Figure 4. The height *dz* of each discrete element depends on the height range of the CWE and the number of discrete elements, which can be expressed as follows:

(19) $$dz=\frac{Z_{max}-Z_{min}}{N_{z}}$$

where *Z_max_* and *Z_min_* denote the maximum and minimum Z-coordinates of the CWE zone, and *N_z_* represents the number of discrete elements. In five-axis micro-milling, the tangential, radial, and axial cutting forces acting on the infinitesimal element of the cutting edge located at the axial height *z* on the *j*-th cutting tooth can be formulated as follows:

(20) $$\left\{\begin{array}{c} dF_{t,j}(t,z)=\delta_{j}(t,z)\left(K_{te}dS+K_{tc}h(t,z)db\right)\\ dF_{r,j}(t,z)=\delta_{j}(t,z)\left(K_{re}dS+K_{rc}h(t,z)db\right)\\ dF_{a,j}(t,z)=\delta_{j}(t,z)\left(K_{ae}dS+K_{ac}h(t,z)db\right) \end{array}\right.$$

where *K_tc_*, *K_rc_*, and *K_ac_* are the tangential, radial, and axial cutting force coefficients, *K_t__e_*, *K_r__e_*, and *K_a__e_* are the tangential, radial, and axial edge force coefficients, and *dS* is the length of the infinitesimal element on the cutting edge, which can be expressed as follows:

(21) $$dS=\sqrt{{\left(R\prime (z)\right)}^{2}+{\left(R(z)\right)}^{2}+1}dz$$

where *dz* is the thickness of the element, and *db* is the cutting width of the element, which can be expressed as follows:

(22) $$db=\frac{dz}{\sin\left(\kappa (z)\right)}$$

where *κ*(*z*) is the axial immersion angle.

The unit step function *δ_j_*(*t*, *z*) determines whether the *j*-th tooth at the axial height *z* is cutting, which can be expressed as follows:

(23) $$g\left(\varphi_{j}(t,z)\right)=\left\{\begin{array}{c} 1,if\;\varphi_{st}(z)<\varphi_{j}(t,z)<\varphi_{ex}(z)\\ 0,otherwise \end{array}\right.$$

where *φ_st_*(*z*) and *φ_ex_*(*z*) represent the entry and exit angles of the cutter, respectively, which are determined through the CWE.

After mapping the calculated tangential, radial, and axial forces to the TCS, the following relationship can be obtained:

(24) $$\left[\begin{array}{c} dF_{x,j}(t,z)\\ dF_{y,j}(t,z)\\ dF_{z,j}(t,z) \end{array}\right]=\left[\begin{array}{ccc} -\cos\varphi_{j}(t,z) & -\sin\varphi_{j}(t,z)\sin\kappa (z) & -\sin\varphi_{j}(t,z)\cos\kappa (z)\\ \sin\varphi_{j}(t,z) & -\cos\varphi_{j}(t,z)\sin\kappa (z) & -\cos\varphi_{j}(t,z)\cos\kappa (z)\\ 0 & \cos\kappa (z) & -\sin\kappa (z) \end{array}\right]\left[\begin{array}{c} dF_{t,j}(t,z)\\ dF_{r,j}(t,z)\\ dF_{a,j}(t,z) \end{array}\right]$$

In the TCS, the total cutting force exerted on the tool can be expressed as follows:

(25) $$\left[\begin{array}{c} F_{x}(t)\\ F_{y}(t)\\ F_{z}(t) \end{array}\right]=\sum_{j=1}^{N_{t}}\sum_{m=1}^{N_{z}}\left[\begin{array}{c} dF_{x,j}(t,z)\\ dF_{y,j}(t,z)\\ dF_{z,j}(t,z) \end{array}\right]$$

Here, *N_z_* denotes the number of discrete elements of the CWE zone, and *N_t_* is the number of flutes. For comparison and verification, the cutting force in the TCS is converted to the GCS via coordinate transformation. The transformation process is expressed as follows:

(26) $$\left[\begin{array}{c} F_{X}(t)\\ F_{Y}(t)\\ F_{Z}(t) \end{array}\right]=R_{1}R_{2}\left[\begin{array}{c} F_{x}(t)\\ F_{y}(t)\\ F_{z}(t) \end{array}\right]$$

where *R*_1_ and *R*_2_ have been provided in Equations (3) and (4).

## 3. Results and Discussion

### 3.1. Experiments and Analysis

Five-axis micro-milling experiments were performed on a JDGR200V high-speed machining center. The cutting forces during the machining process were measured using a Kistler 9119A dynamometer. A micro-impeller and a micro-spherical part made of aluminum alloy 7075-T6 were selected as the workpieces. A two-flute cemented tungsten carbide ball-end mill was employed, with a helix angle of 30°, a diameter of 0.6 mm, and a cutting-edge length of 2 mm.

#### 3.1.1. Measurement of Tool Runout

**Figure 5:**
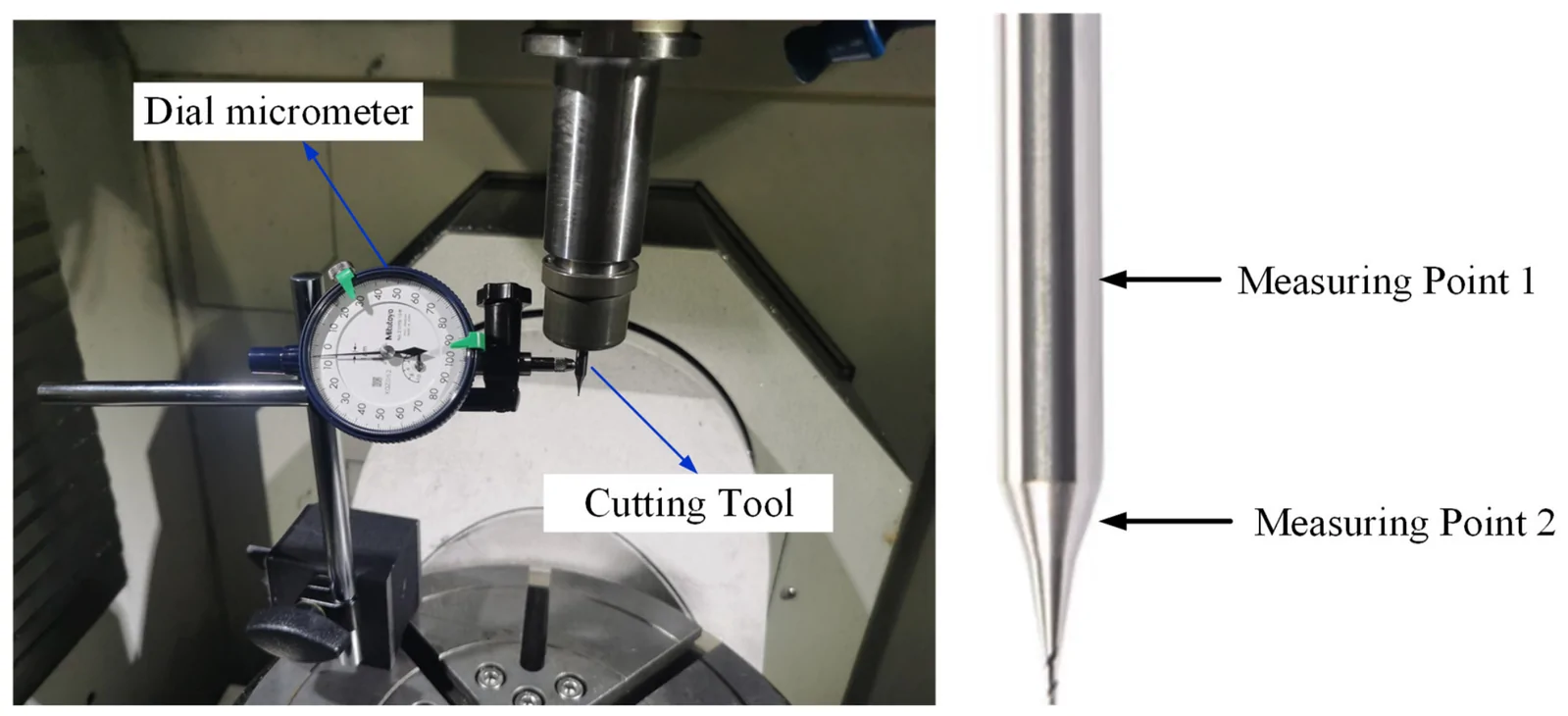
Tool runout measurement setup and measuring points.

**Figure 6:**
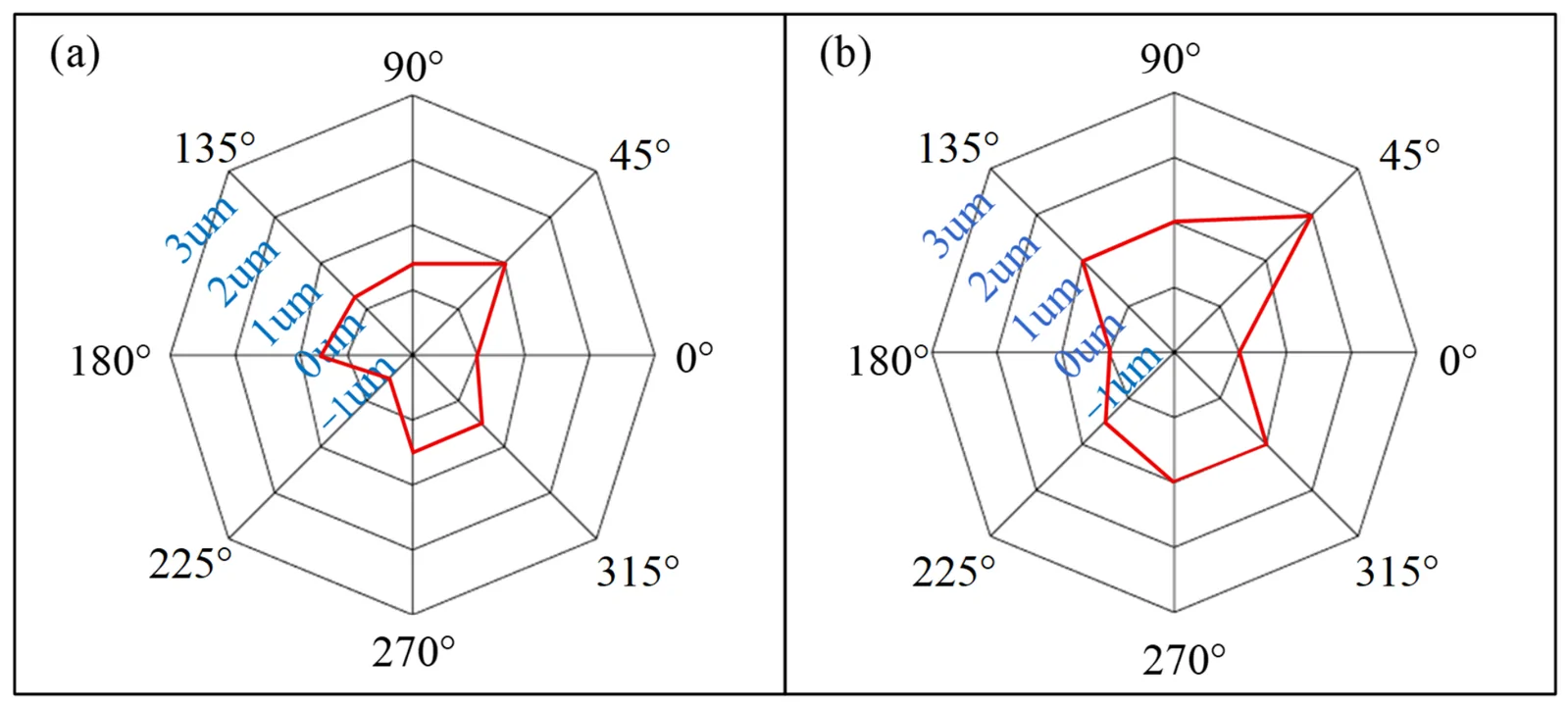
Tool runout measurement results: (**a**) measuring point 1; (**b**) measuring point 2.

To accurately predict the cutting forces in five-axis micro-milling, precise measurement of tool runout is essential. In this study, a dial indicator was employed to measure the tool runout, with the indicator fixed to the machine worktable during the measurement. The measurement setup and points are shown in Figure 5. Measurements were taken at both measuring points shown in Figure 5. During the measurement, the machine spindle was rotated through a full revolution, and the runout length and runout angle were recorded at eight equally spaced angular positions. Based on multiple measurements, the average of the maximum runout lengths observed during each full revolution was taken as the final runout length, and the corresponding angle was adopted as the runout angle. The measurement results are shown in Figure 6, where the average runout length and the corresponding angle are 2 μm and 45°, respectively.

#### 3.1.2. Calibration of Cutting Force Coefficients

The calibration of cutting force coefficients is crucial to accurate force prediction in five-axis micro-milling. Previous studies [22,23,24] have employed linear regression for this purpose and have demonstrated the validity of the resulting coefficients. The same approach is adopted in the present study. For calibration, five sets of experiments were conducted using the parameters listed in Table 1.

To minimize experimental errors, each experiment was repeated three times, and the average values were adopted. The average cutting forces obtained from the experiments are listed in Table 2.

Based on the data in Table 1 and Table 2, the cutting force coefficients and edge force coefficients were determined by linear regression, as shown in Table 3.

#### 3.1.3. Verification of CWE Results

In Section 2.3, the solid modeling method for extracting the CWE in five-axis micro-milling was introduced. To verify the effectiveness of this CWE extraction method for five-axis micro-milling, milling experiments were carried out on an aluminum alloy 7075-T6 block with a micro-end mill. The actual CWE regions after machining were measured using a high-definition industrial camera. The parameters adopted for CWE verification are shown in Table 4, while the comparison between predicted and measured CWE results is presented in Table 5.

It can be observed from Table 5 that the CWE varies with the tool axis inclination angles, which indicates that tool orientation affects the CWE and further influences the five-axis micro-milling forces. On the whole, the CWE extracted based on the solid modeling method shows good agreement with the measured CWE obtained from actual machining. This proves that the solid modeling method is applicable to extracting the CWE in five-axis micro-milling with relatively high accuracy. Accordingly, this method is further employed for CWE extraction during five-axis micro-milling of micro-impeller and micro-spherical part.

It should be noted that, to facilitate comparison with the measured CWE, the CWE in Table 5 is presented in top-view form. In the following discussion, the CWE is instead expressed in terms of the immersion angle to facilitate cutting-force calculation. The immersion-angle representation of the CWE is derived from its top-view form, and both representations of the CWE can characterize the engagement state between the tool and the workpiece.

### 3.2. Experimental Validation of Five-Axis Micro-Milling of Micro-Impeller

**Figure 7:**
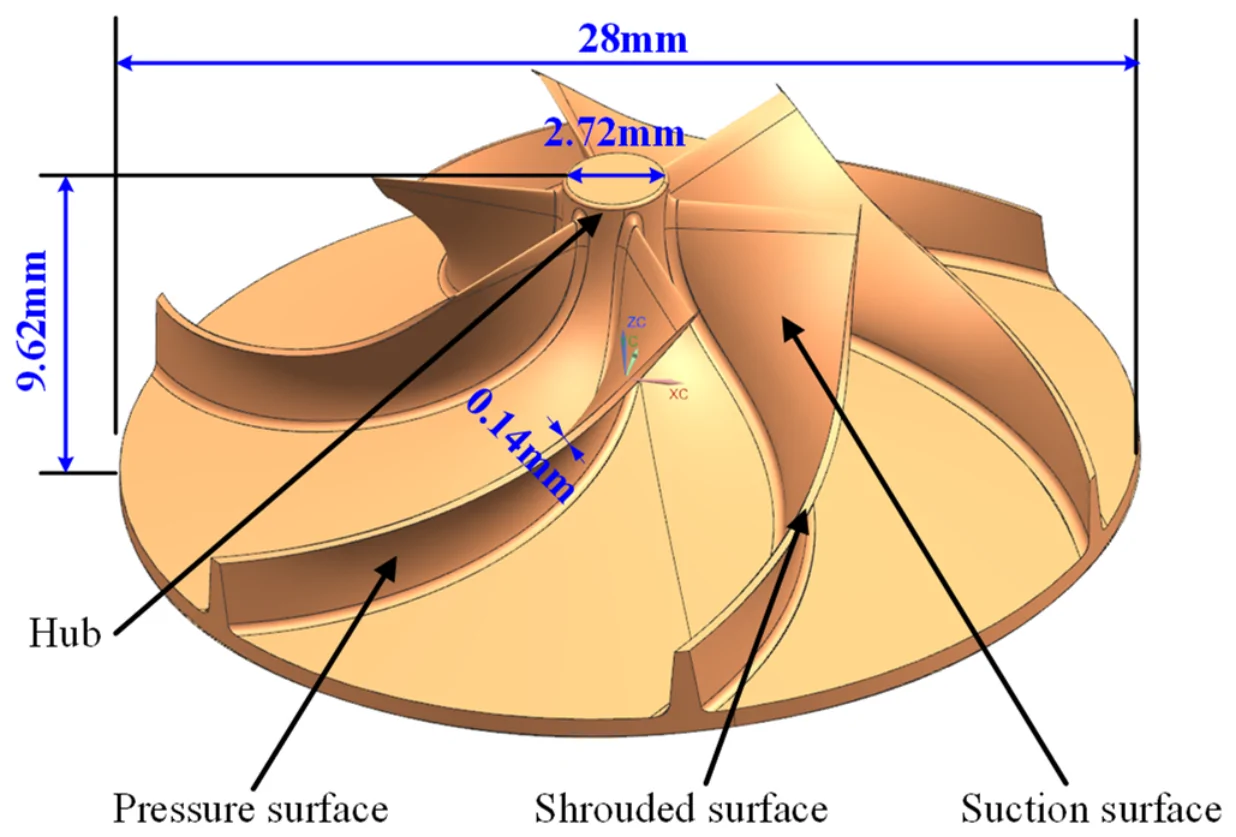
Geometric structure and key dimensions of the micro-impeller.

In this section, the micro-impeller is selected as a typical micro-scale complex curved-surface part to investigate the milling forces in its five-axis micro-milling process. The micro-impeller consists of a hub and multiple blades, of which the blade structure is relatively complex, mainly comprising the pressure surface, shroud surface, and suction surface, and the blade are typically machined via five-axis CNC milling. The main structure and key dimensions of the micro-impeller adopted in this study are shown in Figure 7.

**Figure 8:**
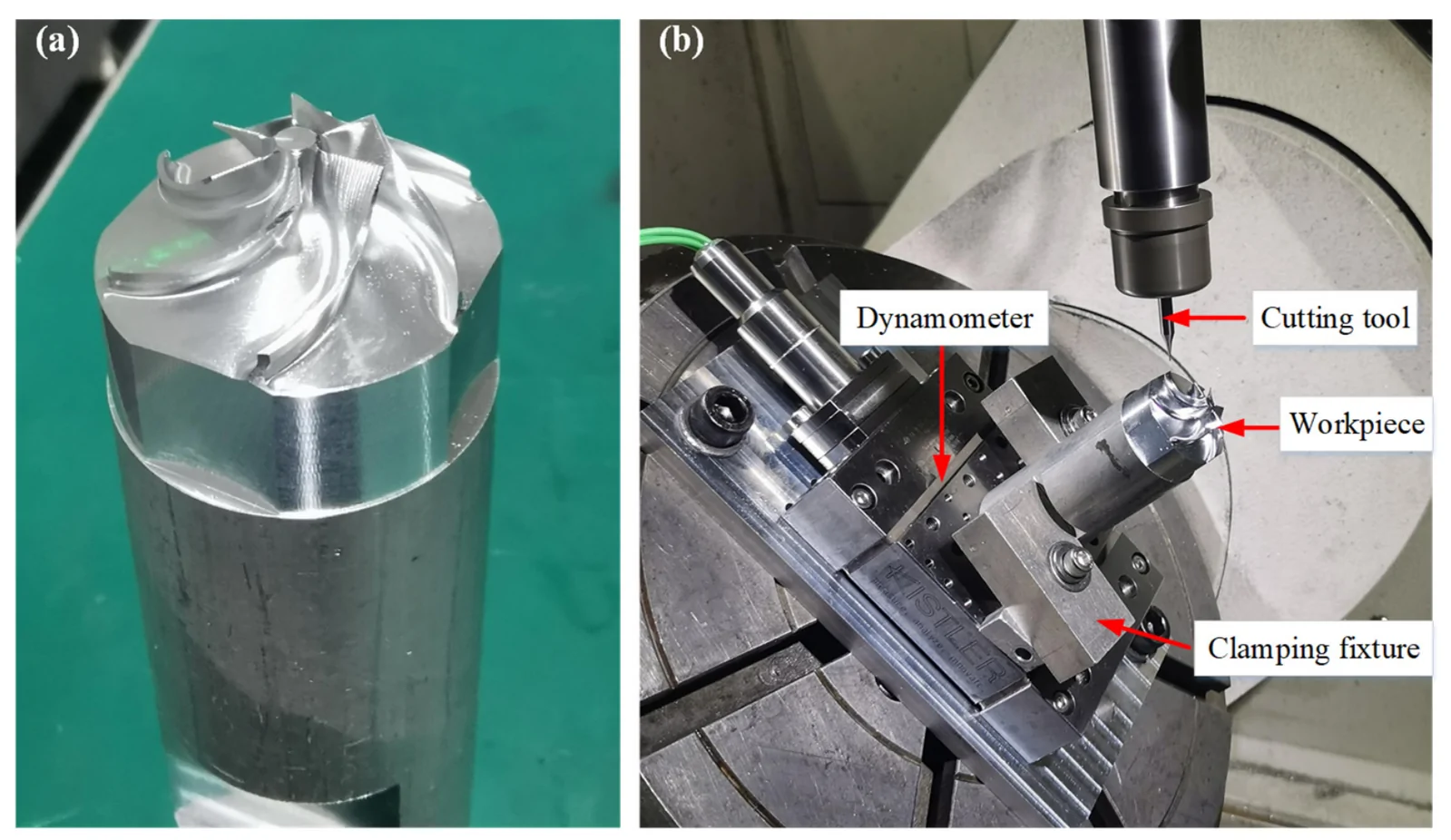
Machining setup. (**a**) The semi-finished micro-impeller. (**b**) The workpiece clamping schematic.

During the machining of micro-impeller blade, five-axis point milling is a commonly adopted method. The roughing and semi-finishing operations are primarily aimed at leaving a uniform allowance for the subsequent finishing process, and their accuracy requirements are generally lower than those of finishing. In contrast, the finishing process has a significant impact on the working performance of the micro-impeller. Therefore, this study focuses on extracting the CWE and predicting milling forces during the finishing of micro-impeller blade. During workpiece clamping, the center point at the bottom of the workpiece was aligned with the rotation axis of the machine’s C-axis. During milling, the spindle speed was 20,000 rpm, the feed rate was 500 mm/min, and dry cutting was employed. The semi-finished micro-impeller and the workpiece clamping schematic are shown in Figure 8.

#### 3.2.1. Extraction of CWE in Five-Axis Micro-Milling of Micro-Impeller Blade

**Figure 9:**
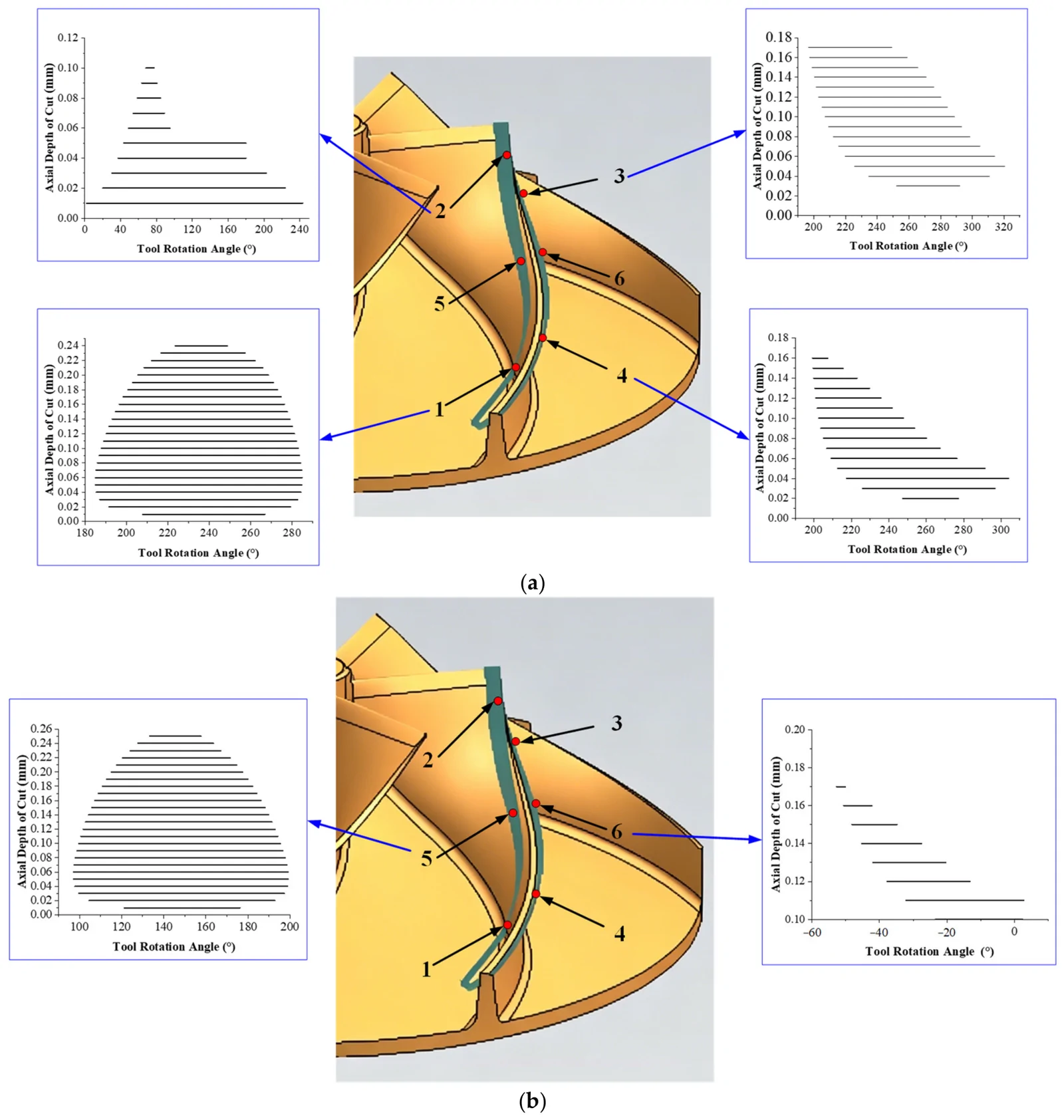
CWE at different CL points during the micro-impeller blade finishing process: (**a**) CWE of the CL points corresponds to the first cut scenario. (**b**) CWE of the CL points corresponds to the following cut scenario. The numbers 1–6 in the figure indicate the CL points 1–6.

During five-axis micro-milling of the micro-impeller, the tool axis inclination angle varies continuously along the tool path, thereby inducing dynamic changes in the CWE. Owing to the greater geometric complexity of micro-impeller blade surfaces, the range of tool axis inclination angle variation is comparatively larger, resulting in more pronounced CWE variability throughout the machining process. Consequently, the CWE is extracted specifically for the finish-machining process of micro-impeller blade. Within each tool path during blade machining, the extraction procedure for CWE geometry remains identical, encompassing two distinct scenarios: the first cut and the following cut. Performing Boolean operations on a large number of cutter location (CL) points in five-axis machining incurs extremely high computational costs, which is a practical limitation of the solid modeling method. To control the computational burden while maintaining accuracy, only the first two tool paths in the blade finishing process are considered in this study, and the corresponding NC code was generated using CAM software. The CWE at the selected CL points was extracted in advance through virtual machining in the CAM environment, although this preprocessing required considerable computation time. Constrained by machining time and data storage requirements, six representative CL points were selected from the two tool paths on a single blade for CWE extraction and cutting force validation. The first four CL points correspond to the first cut, with the CWE illustrated in Figure 9a; the remaining two correspond to the following cut, with the CWE shown in Figure 9b.

As shown in Figure 9, the CWE varies with the CL points during the five-axis finish milling of the micro-impeller blade, thereby affecting the engagement angle range and the actual axial depth of cut. For example, at CL point 5, the axial depth of cut ranges from 0.01 mm to 0.25 mm, and the engagement angle range is approximately 95° to 200°. At CL point 6, the axial depth of cut ranges from 0.11 mm to 0.17 mm, and the engagement angle range is approximately −55° to 5°.

#### 3.2.2. Prediction and Validation of Five-Axis Micro-Milling Forces for Micro-Impeller

**Figure 10:**
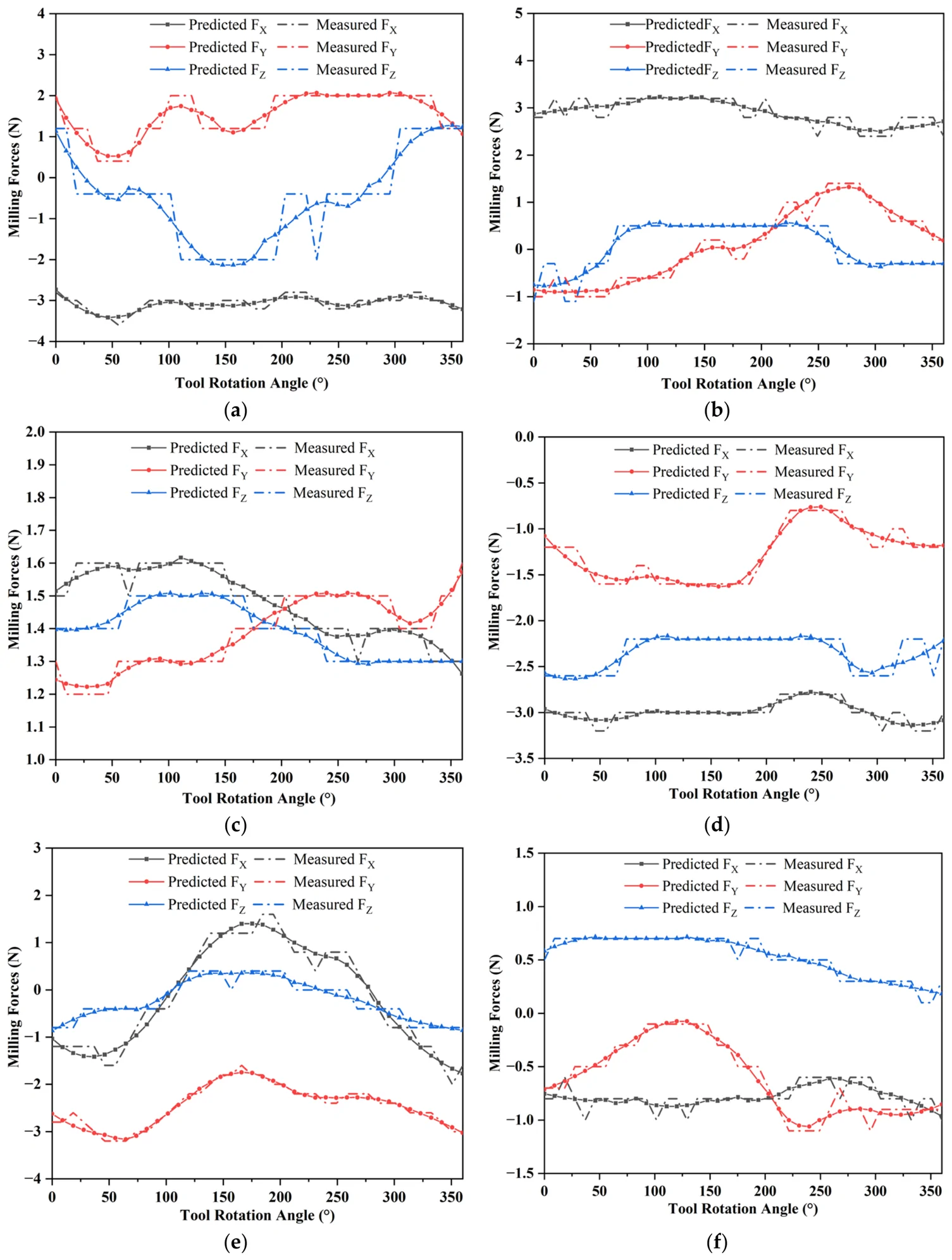
Comparison between predicted and measured milling forces during micro-impeller blade finishing: (**a**) CL point 1. (**b**) CL point 2. (**c**) CL point 3. (**d**) CL point 4. (**e**) CL point 5. (**f**) CL point 6.

Based on the CWE extracted at the aforementioned CL points, and combined with the five-axis micro-milling force prediction model proposed in this study, the milling forces over one spindle revolution at the six CL points were obtained and compared with the experimentally measured milling forces, as shown in Figure 10.

As shown in Figure 10, the milling forces in the X, Y, and Z directions vary continuously with the CL points. Overall, for both the first cut scenario (Figure 10a–d) and the following cut scenario (Figure 10e,f), the predicted cutting forces are in good agreement with the measured values. In Figure 10, the dashed lines denote the milling forces measured by the dynamometer. The long-term constant values are physically measured during the cutting experiments, rather than resulting from signal saturation, low-pass filtering, or any other data processing. The higher constant segments correspond to the stable immersion interval, during which the cutting edge maintains steady contact with the workpiece and the CWE remains essentially unchanged; consequently, the entry and exit immersion angles of the axial disk elements, the instantaneous undeformed chip thickness, and hence the milling force all stay nearly constant, producing the extended plateaus in the measured curves. The lower constant segments correspond to the out-of-cut intervals, in which the cutting edge is not in contact with the workpiece and the measured signal settles at a low constant level. The transitions adjacent to these plateaus reflect the cutter entry and exit stages: during entry, the cutting edge gradually engages the workpiece and the measured force rises with the increasing cutting load, whereas during exit, the edge gradually disengages from the blade and the force decreases accordingly. The predicted forces exhibit the same constant tendency over the corresponding angular intervals.

**Figure 11:**
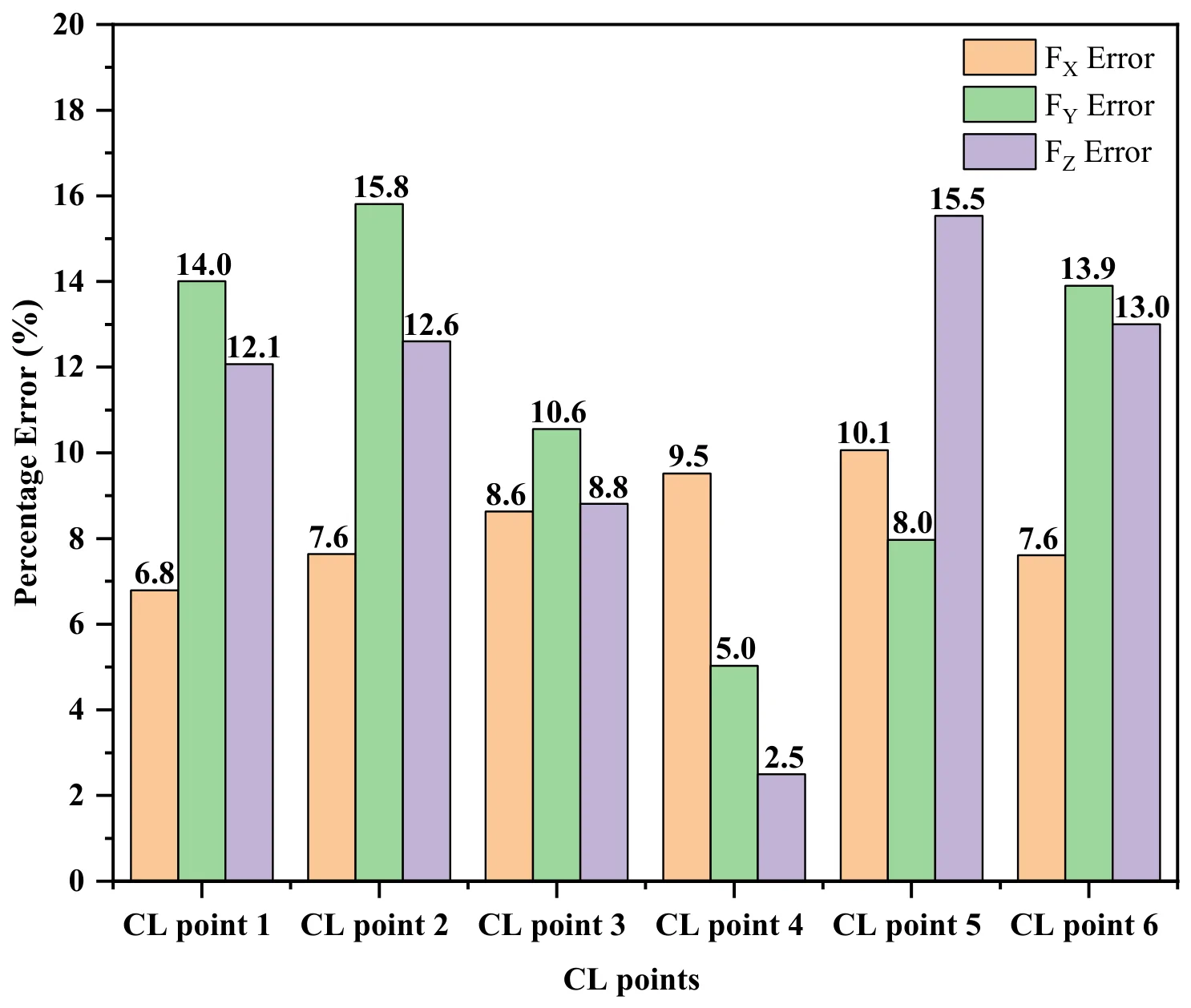
Percentage error of the predicted and measured values at different CL points.

To further verify the proposed milling force prediction model, a percentage error map between the predicted and measured values at the six CL points during the micro-impeller blade finishing process is plotted in Figure 11.

As shown in Figure 11, among the six CL points, the directions in which the maximum and minimum percentage errors of the milling force occur vary from point to point. For instance, at CL points 1, 2, and 3, the largest percentage error occurs in the Y-direction, whereas the smallest occurs in the X-direction; at CL point 5, by contrast, the error is largest in the Z-direction and smallest in the Y-direction. This variation can be attributed to the continuous change in the tool axis orientation across CL points in five-axis micro-milling, which redistributes the cutting force among the X, Y, and Z directions and thus yields direction-dependent percentage errors. Overall, the percentage error between the predicted and measured values of the milling force F_Y_ at CL point 2 is the largest, reaching 15.8%, while that of F_Z_ at CL point 4 is the smallest, at 2.5%. The average percentage errors of the six CL points in the X, Y, and Z directions are 8.4%, 11.2%, and 10.8%, respectively.

The prediction errors are attributed to four factors: (i) Dry cutting: no coolant was used, and the resulting friction, temperature rise, and material adhesion at the tool–workpiece interface were not modeled, constituting a systematic error source. (ii) Vibration: the small tool diameter and high spindle speed rendered the process susceptible to vibration, which perturbed the instantaneous chip thickness and introduced dynamic force components beyond the static model’s capability. (iii) Tool wear: progressive flank wear altered the cutting-edge geometry and contact conditions, causing the actual force to drift over time, whereas the model retained an unworn-tool state. (iv) Calibration uncertainty: the force coefficients were regressed from only five slot milling tests, and errors were inevitably introduced when predicting the cutting forces at CL points with different tool orientations.

### 3.3. Experimental Validation of Five-Axis Micro-Milling of Micro-Spherical Part

#### 3.3.1. Extraction of CWE in Five-Axis Micro-Milling of Micro-Spherical Part

**Figure 12:**
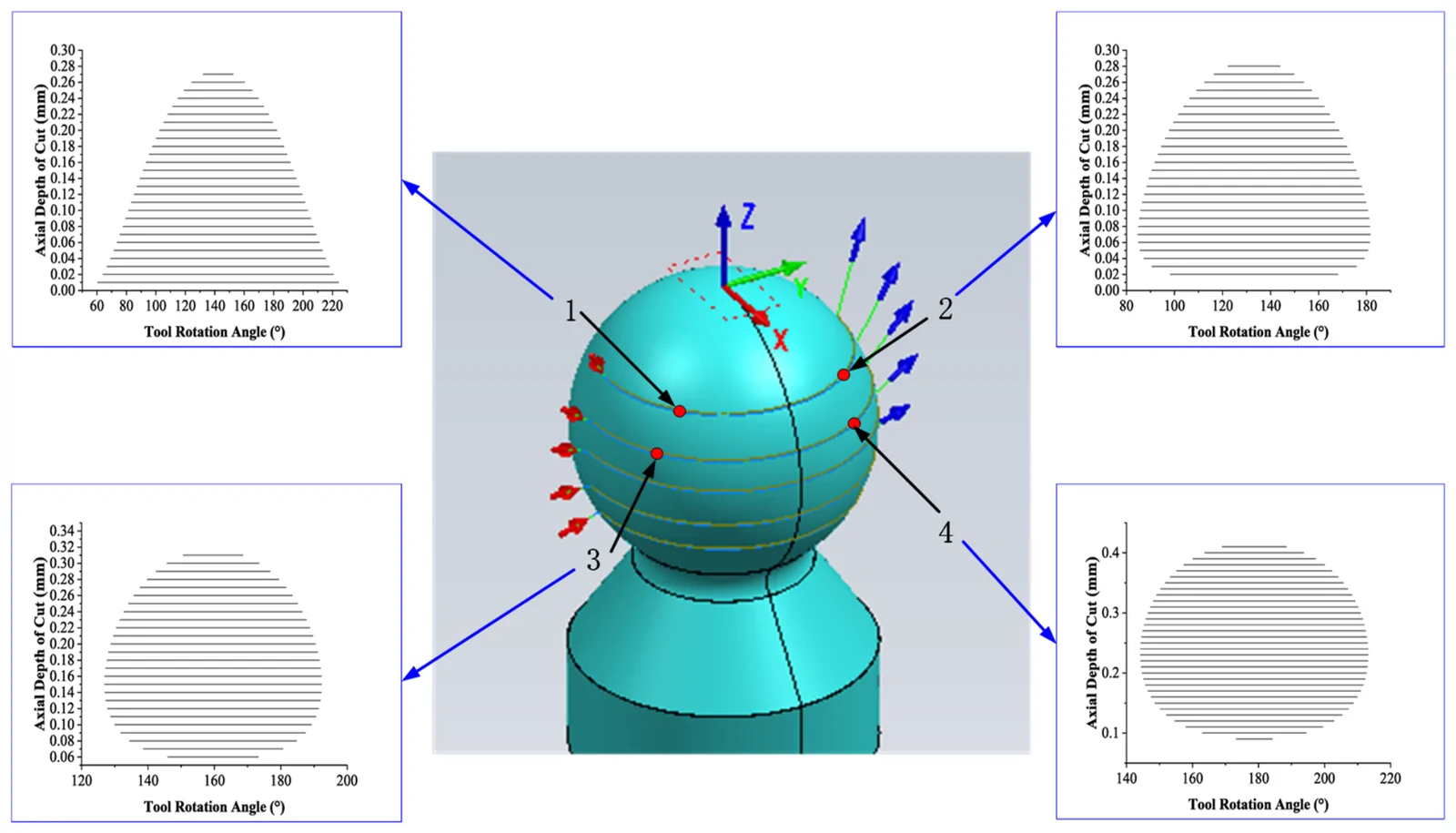
CWE at different CL points during the micro-spherical part micro-milling. The numbers 1–4 indicate CL points 1–4, respectively. The arrows represent the tool axis vector directions along the tool path during hemispherical surface machining, with red arrows for tool entry and blue arrows for tool exit.

To further verify the reliability of the proposed model, five-axis micro-milling experiments were conducted on a micro-spherical part with a diameter of 28 mm. Multiple tool paths were first planned along different latitudes of the spherical part. Since the tool paths along different latitudes of the sphere are essentially identical in shape and pattern, and considering the considerable computational burden, four cutter location (CL) points at different longitudes were selected from the first two tool paths, and the corresponding CWE regions were extracted for the subsequent calculation of micro-milling forces. The CWE regions at the selected CL points are shown in Figure 12.

As shown in Figure 12, during the five-axis micro-milling of the micro-spherical part, the CWE varies at different CL points, which consequently leads to variable cutting forces at these points.

#### 3.3.2. Prediction and Validation of Five-Axis Micro-Milling Forces for Micro-Spherical Part

**Figure 13:**
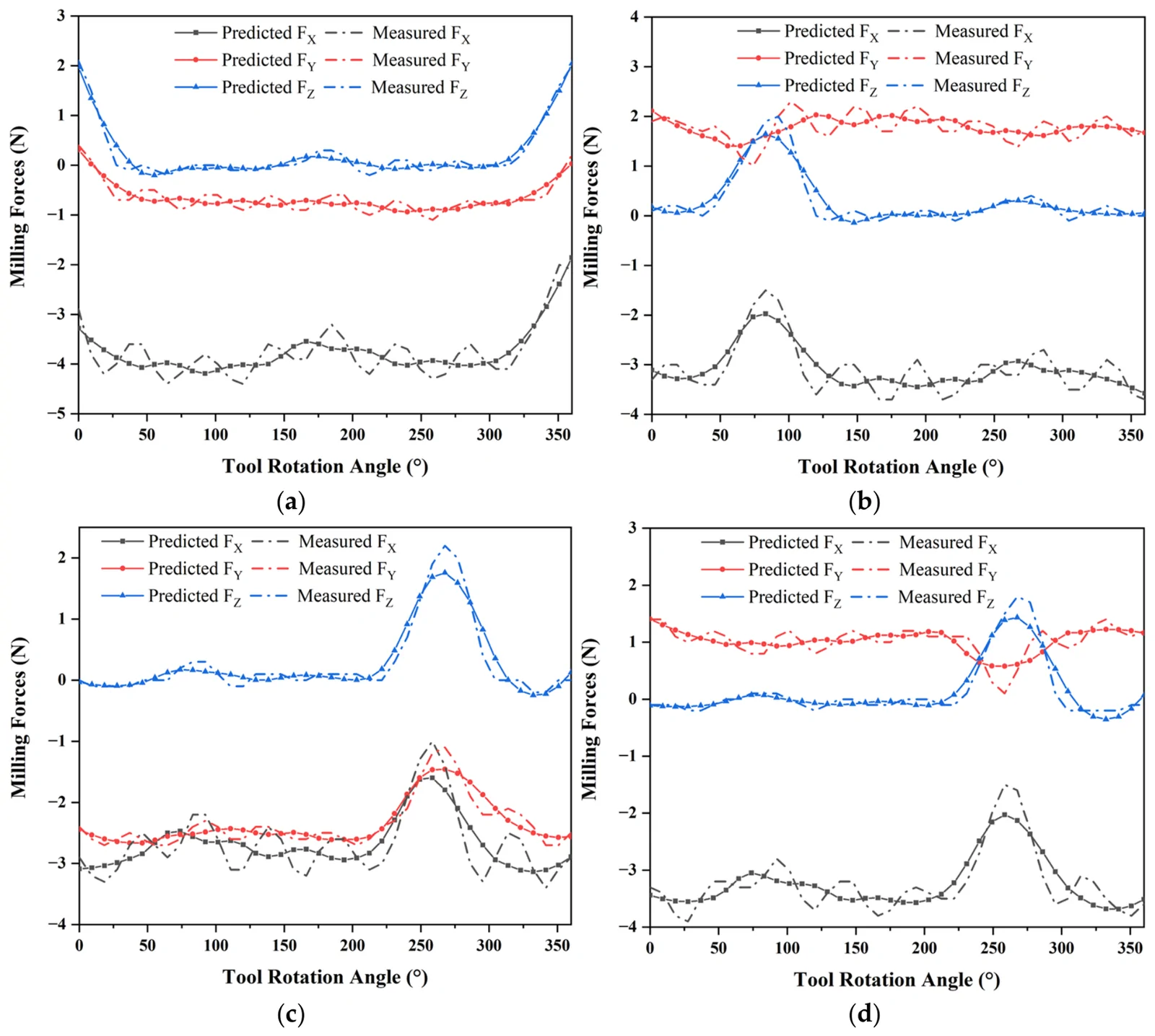
Comparison between predicted and measured milling forces during micro-spherical part milling. (**a**) CL point 1. (**b**) CL point 2. (**c**) CL point 3. (**d**) CL point 4.

Based on the extracted CWE, the milling forces within one revolution at the four CL points were predicted and compared with the experimentally measured milling forces, as shown in Figure 13.

As illustrated in Figure 13, the predicted milling forces in the X, Y, and Z directions show good agreement with the measured values at different CL points. This indicates that the proposed method can accurately predict the milling forces in five-axis micro-milling for both micro-impeller blades and micro-spherical parts, further verifying the reliability of the proposed method. In addition, constant force plateaus similar to those observed in the micro-impeller blade milling (Figure 10) are also observed in the measured curves.

**Figure 14:**
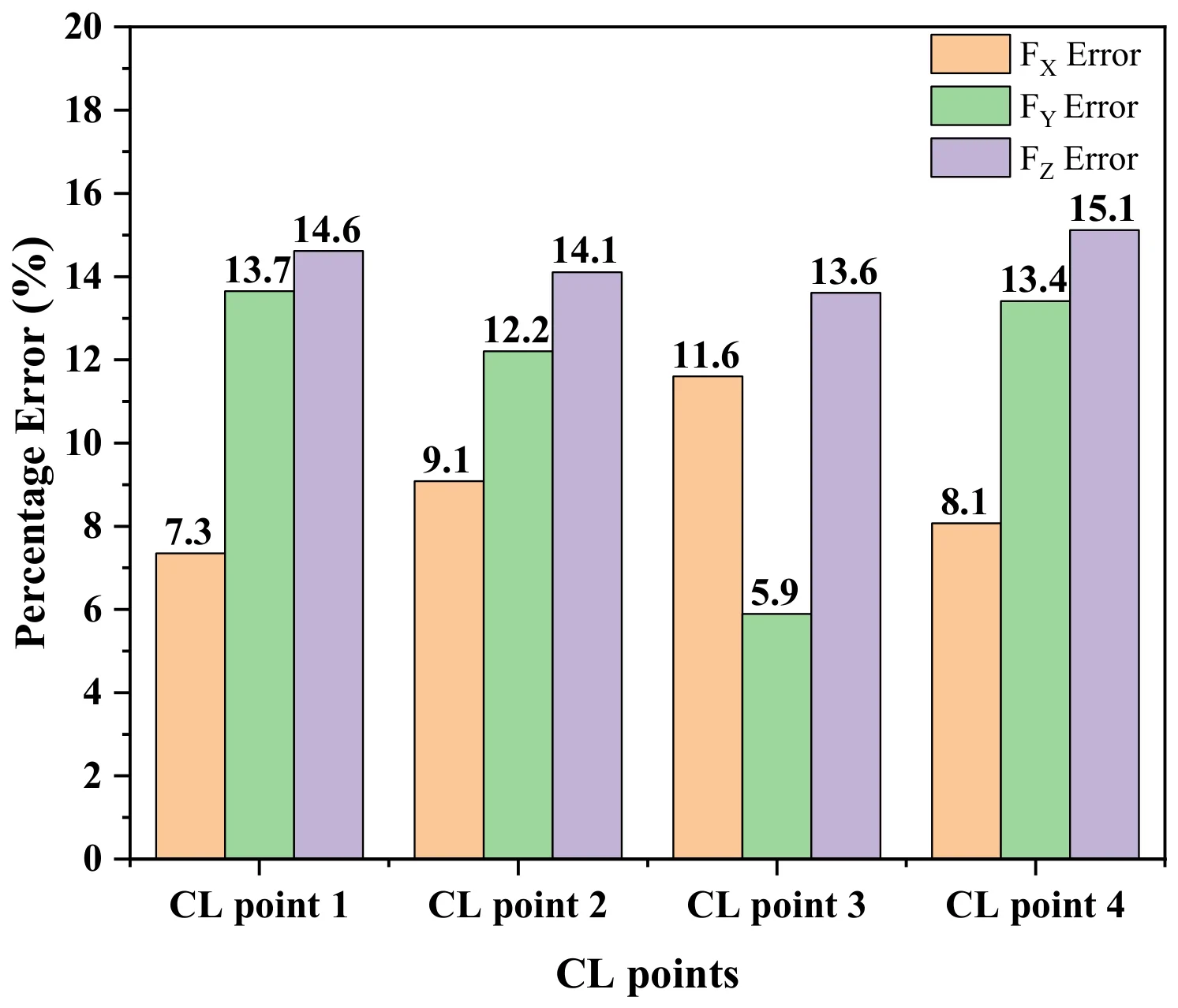
Percentage error of the predicted and measured values at different CL points for micro-spherical part.

To quantitatively evaluate the accuracy of the proposed method, the percentage errors between the predicted and measured milling forces at the four CL points are obtained, as shown in Figure 14.

As shown in Figure 14, the maximum percentage error of 15.1% occurs for the Z-direction milling force F_Z_ at CL point 4, whereas the minimum percentage error of 5.9% is observed for the Y-direction milling force F_Y_ at CL point 3. The average percentage errors of the different CL points in the X, Y, and Z directions are 9.0%, 11.3%, and 14.4%, respectively. It can be seen from Figure 14 that although the discrepancies between predicted and measured values in the X-direction are more noticeable than those in the other two directions, the X-direction cutting force has a larger amplitude, resulting in a smaller average percentage error.

In Ref. [24], Yi et al. predicted the milling forces during the micro-milling of arc-shaped thin-walled parts; the average errors between the predicted and measured micro-milling forces in the X, Y, and Z directions were 12.427%, 11.801%, and 9.005%, respectively. Comparison with Yi et al.’s work in terms of milling-force percentage errors shows that the prediction errors of the proposed method are comparable to those reported in their work, while the workpiece geometry in this work is more complex and the tool orientation and CWE vary more intricately during machining. These findings verify the accuracy of the developed model for five-axis micro-milling of micro-scale curved-surface parts.

The above analysis indicates that although there are certain percentage errors between the predicted and measured values, the predicted values are all within acceptable ranges and can capture the variation trend of the milling forces during the machining process, thereby validating the effectiveness of the model proposed in this study.

## 4. Conclusions

This study aims to predict the milling force in the five-axis micro-milling process. First, the CWE was extracted using a solid modeling approach, and a five-axis micro-milling force prediction model was developed based on the differential element method. Subsequently, the accuracy of the predicted milling forces was validated through five-axis micro-milling experiments on a micro-impeller and a micro-spherical part. The main conclusions of this study are summarized as follows:

(1) Multiple coordinate systems were established for five-axis micro-milling, and the transformation relationships among them were derived. The CWE at different tool axis orientations was extracted using the solid modeling method, and the accuracy of the CWE results was verified experimentally.

(2) An instantaneous undeformed chip thickness (IUCT) model for micro-milling was developed by taking into account the tool runout, the elastic recovery of the machined surface, and the minimum chip thickness. Considering that the local radius of a ball-end mill varies with the axial height, this IUCT model was further extended to the ball-end mill, and a five-axis micro-milling force prediction model was constructed on this basis.

(3) To validate the effectiveness of the proposed model, five-axis micro-milling experiments were conducted for the finish machining of the micro-impeller blade and micro-spherical part. The milling force at each CL point was predicted using the proposed model and compared with the experimental results. The results demonstrate that the predicted five-axis micro-milling forces agree well with the measured values in both the first-cut and following-cut scenarios.

(4) The results indicate that, for the micro-impeller blade, the average percentage errors in the X, Y, and Z directions at all selected CL points are below 11.2%. For the micro-spherical part, the average percentage errors of the selected CL points in the X, Y, and Z directions are less than 14.4%. Comparison with the existing literature in terms of milling-force percentage errors shows that the milling force prediction errors of the proposed method are comparable to those reported in the referenced study, while the workpiece geometry in this work is more complex and the tool orientation and CWE vary more intricately during machining. These findings verify the accuracy of the developed model for five-axis micro-milling of micro-scale curved-surface parts.

Although the effectiveness of the present model has been validated, it has several limitations regarding its application scope. The model is applicable to other metallic materials; however, the cutting force coefficients must be recalibrated for each specific workpiece material. The model was developed on the basis of ball-end mills, and its geometric framework requires corresponding modification before extension to other cutter types, such as taper or toroidal end mills. Furthermore, the effects of tool wear and tool deflection are not considered in the current model; incorporating these factors into the model constitutes an important direction of our future work. Finally, the solid modeling method adopted in this study introduces a heavy computational cost during CWE extraction, calling for more efficient extraction algorithms in future research.

## Figures and Tables

**Table 1:** Experimental parameters.

Test No.	Cutter Radius (mm)	Spindle Speed (rpm)	Axial Depth of Cut (mm)	Feed per Tooth (mm/tooth)
1	0.3	20,000	0.3	0.002
2	0.3	20,000	0.3	0.004
3	0.3	20,000	0.3	0.006
4	0.3	20,000	0.3	0.008
5	0.3	20,000	0.3	0.01

**Table 2:** Experimental results of average milling forces.

Test No.	Average Milling Forces (N)		
	F_X_	F_Y_	F_Z_
1	0.8610	1.6997	0.5940
2	1.3597	2.7304	1.2413
3	1.833	3.6116	1.7858
4	1.966	4.2707	2.1142
5	2.119	4.8946	2.8787

**Table 3:** Cutting force coefficients.

Cutting Force Coefficients	Value (N/mm^2^)	Edge Force Coefficients	Value (N/mm^2^)
$K_{rc}$ (N/mm^2^)	1040.29	$K_{re}$ (N/mm^2^)	3.6201
$K_{tc}$ (N/mm^2^)	2643.37	$K_{te}$ (N/mm^2^)	5.5626
$K_{ac}$ (N/mm^2^)	−1424.82	$K_{ae}$ (N/mm^2^)	−0.3002

**Table 4:** Process parameters for CWE verification.

Test No.	Cutting Depth (mm)	Lead Angle (°)	Tilt Angle (°)
1	0.2	0	0
2	0.2	0	15
3	0.2	15	0
4	0.2	15	15
5	0.2	0	−15

**Table 5:** Comparison of the predicted and measured CWE. The red lines delineate the CWE boundaries, and the accuracy of the prediction method is verified by comparing whether the regions outlined by the red lines in the two columns coincide.

Test No.	Top-View of the CWE	Measured CWE
1	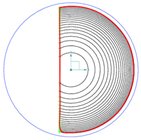	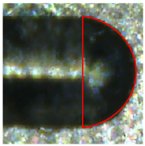
2	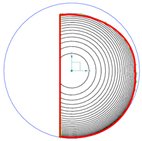	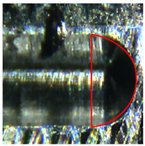
3	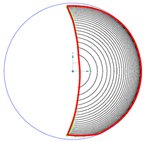	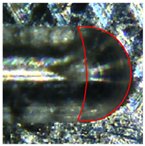
4	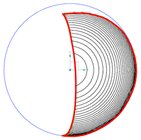	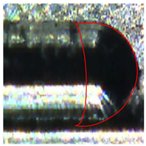
5	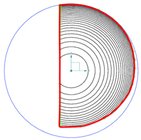	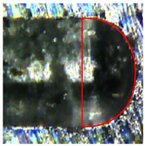

## Data Availability

The data that support the findings of this study are available from the corresponding author upon reasonable request.

## Abbreviations

The following abbreviations are used in this manuscript:

CWE	Cutter Workpiece Engagement
IUCT	Instantaneous Uncut Chip Thickness
CL	Cutter Location
GCS	Global Coordinate System
WCS	Workpiece Coordinate System
FCN	Feed-Cross Feed-Normal coordinate system
TCS	Tool Coordinate System
